# Parental body mass index and offspring childhood body size and eating behaviour: A structural equation modelling analysis in the Norwegian Mother, Father and Child Cohort Study

**DOI:** 10.1371/journal.pmed.1005094

**Published:** 2026-06-23

**Authors:** Tom A. Bond, Tom A. McAdams, Nicole M. Warrington, Laurie J. Hannigan, Espen Moen Eilertsen, Ziada Ayorech, Fartein A. Torvik, George Davey Smith, Deborah A. Lawlor, Eivind Ystrom, Alexandra Havdahl, David M. Evans

**Affiliations:** 1 Frazer Institute, The University of Queensland, Brisbane, Australia; 2 MRC Integrative Epidemiology Unit at the University of Bristol, Bristol, United Kingdom; 3 Population Health Sciences, Bristol Medical School, University of Bristol, Bristol, United Kingdom; 4 Department of Epidemiology and Biostatistics, Imperial College London, London, United Kingdom; 5 Social, Genetic and Developmental Psychiatry Centre, Institute of Psychiatry, Psychology and Neuroscience, King’s College, London, United Kingdom; 6 PROMENTA Research Center, Department of Psychology, University of Oslo, Oslo, Norway; 7 Institute for Molecular Bioscience, University of Queensland, Brisbane, Australia; 8 K.G. Jebsen Center for Genetic Epidemiology, Department of Public Health and Nursing, NTNU, Norwegian University of Science and Technology, Trondheim, Norway; 9 Research Department, Lovisenberg Diakonale Hospital, Oslo, Norway; 10 PsychGen Centre for Genetic Epidemiology and Mental Health, Division for Public Health and Prevention, Norwegian Institute of Public Health, Oslo, Norway; 11 Centre for Fertility and Health, Norwegian Institute of Public Health, Oslo, Norway; 12 Centre for Research on Equality in Education, Faculty of Educational Sciences, University of Oslo, Oslo, Norway; 13 Department of Child Health and Development, Norwegian Institute of Public Health, Oslo, Norway; 14 Nic Waals Institute, Lovisenberg Diakonale Hospital, Oslo, Norway

## Abstract

**Background:**

The intergenerational transmission of obesity-related traits could propagate an accelerating cycle of obesity, if parental adiposity causally influences offspring adiposity. The extent to which intergenerational obesity associations are due to such causal effects, as opposed to genetic confounding (inheritance), is unclear. We aimed to establish whether associations between parental peri-pregnancy body mass index (BMI) and offspring birth weight (BW), BMI until 8 years of age, and 8-year-old eating behaviour are due to genetic confounding.

**Methods and findings:**

Data were from the Norwegian Mother, Father and Child Cohort Study, a prospective population-based birth cohort born between 1999 and 2009 at 50 out of 52 hospital maternity units in Norway. We compared the strength of the associations of maternal pre-pregnancy BMI versus paternal BMI during pregnancy, with offspring outcomes including birth weight and BMI assessed between age 6 months and 8 years of age, and appetite-related eating behaviour traits assessed at age 8 years via the Child Eating Behaviour Questionnaire (CEBQ), adjusting for potential confounders including parity, parental/grandparental language group and parental age, smoking, education and income). We then used an extended children of twins structural equation model (SEM) to quantify the extent to which associations were due to genetic confounding. Up to 85,866 children (51.3% male) were included in linear regression models, whereas SEM models included up to 50,999 children. Maternal BMI was more strongly associated than paternal BMI with offspring BW, but the maternal-paternal difference decreased for offspring BMI after birth. Greater parental BMI was associated with obesity-related offspring eating behaviours. SEM results indicated that genetic confounding did not explain the association between parental BMI and offspring BW, but explained the majority of the association with offspring BMI from 6 months onwards. For 8-year BMI, genetic confounding explained 79% (95% CI [62, 95]; *p* = 1.9 × 10^−12^) of the covariance with maternal BMI and 94% (95% CI [72, 113]; *p* = 2.7 × 10^−14^) of the covariance with paternal BMI. Limitations of this study include selective recruitment and attrition, potential bias due to parental assortative mating, and that findings may not generalise beyond high-income country settings with high obesity prevalence.

**Conclusions:**

We found strong evidence that parent–child BMI associations may primarily be due to genetic confounding. When considered alongside prior evidence, this finding may argue against a strong causal effect of maternal or paternal adiposity on childhood adiposity via intrauterine or periconceptional mechanisms.

## Author summary

**Why was this study done?:**

- Greater parental body mass index (BMI) is associated with childhood obesity-related traits in the offspring, including weight, BMI and appetite-related eating behaviours
- We aimed to explore whether these associations are due to genetic inheritance, as opposed to other mechanisms such as a causal effect of parental BMI on offspring obesity traits

**What did the researchers do and find?:**

- We found that in a sample of up to 85,866 Norwegian children born between 1999 and 2009, there were not large differences in the associations of the mother’s and father’s BMI with the child’s BMI at age 2–8 years
- We then applied statistical structural equation modelling to participants in the sample whose relatedness was known, and found that the majority of the association between parental BMI and offspring BMI from age 6 months to 8 years was explained by genetic inheritance

**What do these findings mean?:**

- When considered alongside previous studies, our results suggest that because the associations between parental BMI and offspring childhood BMI may mostly be due to genetic inheritance, reductions in parental BMI may not cause reductions in childhood BMI
- Potential limitations of this study include selection bias and bias due to correlations between the parents’ BMI, and the findings may not generalise to lower income countries where there are lower obesity rates

## Abstract 3

In a prospective study, Tom Bond and colleagues investigate whether associations between parental peri-pregnancy body mass index (BMI) and offspring birth weight (BW), BMI until 8 years of age and 8-year old eating behaviour are due to genetic inheritance in a population-based birth cohort from Norway.

## Introduction

The worldwide epidemic of child and adolescent overweight/obesity will have enormous health and financial costs [1], and appears to be intractable in the face of current preventive strategies, with prevalence recently estimated at 30.2% in high income countries [2]. An obesogenic environment is now widespread in many countries, encompassing numerous biological and social determinants of nutrition and physical activity. However, because childhood obesity develops early in life [3] and runs in families [4], effective preventive interventions are increasingly sought which target the parents [5]. The positive observational association between parental body mass index (BMI) and offspring adiposity in childhood is well replicated [4,6], but the mechanisms driving this association remain unknown. If greater maternal or paternal BMI causes greater offspring BMI via prenatal or intrauterine developmental mechanisms, a vicious cycle could amplify BMI through successive generations and be a major driver of the obesity epidemic [7]. It is therefore crucial to establish why parental BMI is associated with offspring childhood BMI.

**Fig 1:**
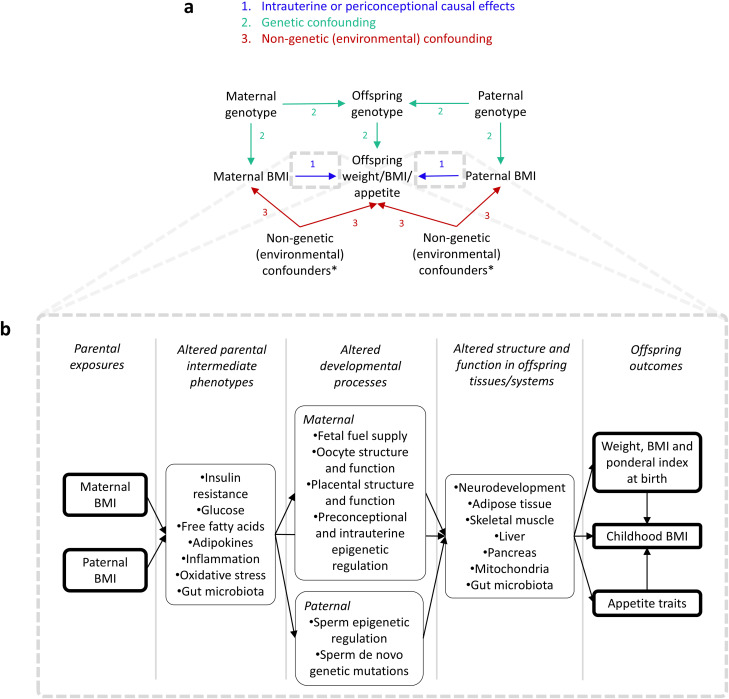
Plausible mechanisms for associations between parental BMI and offspring weight, BMI and appetite traits. **(a)** Directed acyclic graph (DAG) showing three plausible mechanisms for the associations. *We define non-genetic (environmental) confounding as confounding that does not involve the offspring’s own genotype (non-genetic confounding could therefore still involve parental genetic effects, i.e., effects of parental genotype on offspring outcomes independently of offspring genotype, via the offspring’s environment). Examples of non-genetic confounding include shared familial socioeconomic position, confounding effects of parental exposures such as smoking (including via intrauterine and postnatal growth patterns), or parental influences on offspring postnatal food intake and physical activity behaviours. To the extent that postnatal parental BMI per se causally influences offspring outcomes after birth (e.g., by influencing feeding- and exercise-related parenting practices), this would constitute a postnatal causal effect rather than non-genetic confounding. **(b)** Conceptual diagram showing putative biological mechanisms by which parental BMI could have intrauterine or periconceptional causal effects on offspring outcomes. Arrows denote potential causal effects, bold outlined boxes denote variables analysed in the present study. The intent is to non-exhaustively show some key variables and relationships that have been hypothesised in the literature.

Several mechanisms are plausible (Fig 1A). Higher parental BMI could cause higher offspring adiposity through pre-conception and/or intrauterine developmental mechanisms (the developmental overnutrition hypothesis) [8–11], with some authors advocating that interventions to maintain women’s preconception BMI at a healthy level be used as a means to reduce offspring adiposity [6,8,12]. As well as putative effects of maternal BMI during early development, paternal BMI could influence offspring adiposity via effects on the sperm and seminal fluid, including epigenetic alterations and DNA damage [13,14]. Because adiposity is highly heritable across the life course [15], genetic confounding (via the inheritance of parental genetic alleles by the offspring) could result in intergenerational BMI associations. Non-genetic (environmental) confounding, for example, via shared familial socioeconomic position or parental influences on offspring postnatal food intake and physical activity behaviours, could also contribute to these associations [16].

Numerous animal studies purport to provide evidence in favour of the developmental overnutrition hypothesis [17]. Potential biological mechanisms, many involving epigenetic processes [8], have been elucidated (Fig 1B) [10,17], including a putatively key role for the programming of offspring appetite via energy homeostasis brain networks [18]. In humans, child appetite traits are associated with the child’s own BMI [19], and with maternal overweight/obesity [20]. However, whether developmental programming of adiposity and appetite occurs in humans remains unclear. Mendelian randomisation (MR) [21], sibling studies [22,23] and paternal negative exposure control studies [24,25] suggest that familial confounding (either genetic or non-genetic) may be an important cause of parent–child BMI associations. However, such associations are generally unchanged on adjustment for measured variables [26], leaving the specific confounders unidentified. We have shown previously that genome-wide genetic variants explain a large proportion of parent–child BMI associations, implicating genetic factors as an important possible confounder [26]. However, no previous studies have explicitly quantified genetic confounding.

We aimed to establish whether associations between peri-pregnancy parental BMI and offspring birth weight, childhood BMI and appetite-related eating behaviours are due to genetic confounding. We first compared the strength of the associations of maternal versus paternal BMI with offspring outcomes, which are likely to be similarly strong if they are primarily due to genetic confounding. We then applied a genetically informed structural equation model (SEM) to a population-based sample of twins, siblings and half siblings, and their children, to quantify the relative importance of genetic confounding versus other mechanisms in underpinning intergenerational associations. Based on prior evidence [21–27] we hypothesised that genetic confounding would not explain the associations of parental BMI with offspring birth weight, but would be a major driver of associations with offspring childhood BMI. There is strong evidence that BMI in both childhood and adulthood are highly heritable [28–30], and have a high genetic correlation [31], therefore it is highly plausible that genetic confounding is the main cause of parent–child BMI associations. In this case, targeting interventions towards isolated reduction of parental BMI, without altering the offspring’s postnatal environment, may be insufficient to achieve large reductions in the offspring’s childhood obesity risk.

## Methods

### Study design and participants

This study is reported as per the STROBE guidelines (S1 STROBE checklist). Our study did not follow a formal prospective analysis plan, but all analyses were planned prior to analysing the data (aside from the exploratory analyses described in Methods and presented in Information P in S1 Appendix). We analysed data from the Norwegian Mother, Father and Child Cohort Study (MoBa; described in detail elsewhere [32]), a prospective population-based birth cohort conducted by the Norwegian Institute of Public Health, and used data from the Medical Birth Registry of Norway (MBRN), a national health registry containing information about all births in Norway [33]. Pregnant women were recruited at 50 out of 52 hospital maternity units in Norway, on attendance of a routine antenatal ultrasound scan offered to all Norwegian women at around 17 weeks of gestation. Forty-one percent of invitees participated, resulting in a total sample of around 114,500 children born between 1999 and 2009, along with around 95,200 mothers and 75,200 fathers. We used version 11 of the quality-assured data files released for research in 2018, and analysed only live-born offspring. Flowcharts detailing sample selection are presented in Information A in S1 Appendix. The establishment of MoBa and initial data collection was based on a license from the Norwegian Data Protection Agency and approval from The Regional Committees for Medical and Health Research Ethics. The MoBa cohort is currently regulated by the Norwegian Health Registry Act. The current study was approved by The Regional Committees for Medical and Health Research Ethics (REK 2013/863). All participants provided written informed consent.

### Exposures and outcomes

The exposures were maternal pre-pregnancy BMI and paternal BMI during pregnancy, calculated from weight and height reported by the parents at the first study questionnaire (around 17 weeks gestation). Maternal height and pre-pregnancy weight were reported by the mothers, and paternal weight and height were reported by the fathers (for 35% of measurements), or by the mothers when paternal report was unavailable (Pearson’s *r* = 0.98 between maternally and paternally reported paternal weight and height).

Offspring outcomes included birth weight and BMI assessed between age 6 months and 8 years, and appetite-related eating behaviour traits assessed at age 8 years via the CEBQ [34]. Birth weight and length were from the MBRN. Mothers completed regular questionnaires when their children were aged between 6 months and 8 years, from which the child’s weight and height at age 6 months and 1, 2, 3, 5, and 8 years were obtained. Measurements at ages up to 3 years were predominantly from the child’s health card, whereas measurements from 5 years onwards were carried out by the parents. The missingness rate was greater amongst the health card-derived data, and the 5-year questionnaire was not developed before a proportion of children had already turned 5 years, therefore more children had weight and height data available at 8 years than earlier in childhood. In order to maximise statistical efficiency, we also used all available offspring BMI measurements to fit a growth curve, from which we predicted offspring BMI at 1-year intervals between age 1 and 8 years for children with at least three BMI measurements. These fitted BMI values, which we refer to as “predicted BMI”, were used as a supplement to the mother-reported BMI measures described above, enabling comparison of results in an identical (and larger) sample across different ages. Full details of the cleaning of anthropometric data and growth curve fitting are given in Information B in S1 Appendix. As pre-planned secondary outcomes we analysed ponderal index (weight [kg]/length [m]^3^) and BMI at birth, and weight at ages up to 8 years.

The CEBQ is a widely used and validated psychometric instrument for child eating behaviours [34]. At the 8-year questionnaire, mothers completed 5-point Likert scales for 18 of the 35 overall CEBQ items related to their child’s satiety responsiveness, slowness in eating, food responsiveness, fussiness, emotional overeating and emotional undereating. Seventeen CEBQ items, including those related to the scales “desire to drink” and “enjoyment of food”, were unavailable. We calculated the mean item score for each of the six scales, for participants with available data for at least two out of three items per scale. Covariate data were obtained from the MBRN or study questionnaires and are described in Information C in S1 Appendix.

### Linear regression analyses

We fitted linear regression models to explore associations between exposures and outcomes, adjusting when relevant for offspring sex and age at outcome measurement, the other parent’s BMI, and potential non-genetic confounders including maternal parity, parental and grandparental language group (as a proxy for ethnicity) and maternal and paternal characteristics (age, smoking during pregnancy, educational attainment and income). Participants with non-missing values for all relevant variables were included in analyses. To account for non-independence between siblings, we used a linear mixed model with a random intercept at the family level (Information D in S1 Appendix), and a *z*-test was used to test whether associations with maternal and paternal BMI differed in magnitude (Information E in S1 Appendix). For ease of interpretation, exposure and outcome variables were standardised, therefore regression coefficients are interpreted as the average change in the outcome in standard deviation (SD) units per 1 SD increase in the exposure. Because the standard deviation may differ for maternal versus paternal BMI, we also tested for maternal-paternal differences using unstandardised exposures. Offspring BMI from age 5 years onwards was positively skewed (Information F in S1 Appendix) so was natural log transformed, and several CEBQ eating behaviour scores were strongly skewed so were regressed on offspring age and sex followed by rank-based inverse normal transformation of the residuals. We carried out sensitivity analyses including (1) additionally adjusting birth weight models for gestational age at birth; (2) testing for non-linear associations (Information G in S1 Appendix); (3) testing for interaction by offspring sex; (4) testing for maternal BMI-paternal BMI interaction and (5) estimating the maternal BMI-paternal BMI phenotypic correlation. Analyses were carried out in R version 4.0.3 [35].

### Genetically informed structural equation modelling

To quantify the extent to which exposure-outcome associations were due to genetic confounding, we fit an extended children of twins SEM (the Multiple Children of Twins and Siblings [MCoTS] model, described in Information H in S1 Appendix and elsewhere [36]) in a subset of the MoBa sample. An extended pedigree including twins, siblings and half-siblings in both the parental and offspring generations was identified within MoBa using data from the study questionnaires, genotyping, and linkage to the Norwegian Population Registry, the Norwegian Twin Registry and the MBRN [36]. Our MCoTS model partitions the phenotypic covariance between exposures and outcomes into a part due to genetic confounding and a residual part (due to any causal effects and/or non-genetic confounding). Skewed exposure and outcome variables were transformed as for linear regression analyses, with the exception that parental BMI was also natural log transformed given the multivariate normality assumptions of SEM fit via maximum likelihood (i.e., that dependent variables [including parental BMI and offspring outcomes] are multivariate normally distributed). Exposure variables were standardised to give unit variance and zero mean. Outcome variables were standardised (or inverse normalised for eating behaviour variables) within sex strata (or within age and sex strata for child BMI outcomes). Because the variance of exposures and outcome variables was close to one, covariances are approximately equal to Pearson’s correlation coefficients. Classic and extended twin studies suggest the presence of dominance genetic effects and absence of common environmental effects for adult BMI [15], but provide support for common environmental effects on birth weight and child BMI [29,37]. We therefore chose *a priori* to fit an MCoTS model that partitioned parental BMI variance into additive, dominance and non-shared environmental components (an ADE model) and partitioned offspring outcome variance into additive, common environmental and non-shared environmental components (an ACE model). In sensitivity analyses we fit ACE and AE models for parental BMI as well as stratifying analyses by offspring sex, fitting a liability threshold model for untransformed eating behaviour outcomes (Information I, J and K in S1 Appendix), and refitting birth weight models having dropped offspring-generation twins (because monozygotic [MZ] twins may share a placenta and twins have lower birth weight than singletons, which could generate biases). Standard errors and 95% confidence intervals were calculated via bias-corrected bootstrapping of the MCoTS model with 10,000 resamples. SEM were fit in R version 4.0.3 (OpenMx package version 2.18.1) [35,38].

## Results

The number of offspring included in linear regression analyses varied by outcome, from 85,866 (51.3% male), equivalent to 74.9% of the recruited sample, to 30,904 (51.5% male), equivalent to 27.0% of the recruited sample, for analyses of birth weight and 2-year BMI, respectively. Information A in S1 Appendix shows the proportion of the sample with non-missing data for each analysis, and Table 1 shows the characteristics of the study participants. There was statistical evidence for selective attrition, such that the sample used for analyses of 8-year BMI (*n* = 46,620) was more highly educated and had lower obesity prevalence and greater maternal age versus the baseline sample, but the magnitude of such differences was relatively small (Information L in S1 Appendix).

**Table 1:** Characteristics of the parental and offspring generation of study participants (*n* = 85,866).

Variable		Mean	SD	*n*	%^a^	% missing^b^
Parental characteristics						
Maternal BMI (kg/m^2^)		24.1	4.3			11.7
Paternal BMI (kg/m^2^)		25.9	3.3			12.2
Maternal WHO BMI category (kg/m^2^)	<18.5			2,560	3.0	11.7
	18.5–24.9			56,367	65.6	
	25–29.9			18,727	21.8	
	≥30			8,212	9.6	
Paternal WHO BMI category (kg/m^2^)	<18.5			197	0.2	12.2
	18.5–24.9			37,822	44.0	
	25–29.9			39,120	45.6	
	≥30			8,727	10.2	
Parity (number of previous births)	0			38,736	45.1	0.0
	1			30,807	35.9	
	2			12,795	14.9	
	3			2,740	3.2	
	4+			788	0.9	
Maternal age at birth of child (years)	≤19			624	0.7	0.0
	20–24			8,029	9.4	
	25–29			28,245	32.9	
	30–34			33,814	39.4	
	35–39			13,465	15.7	
	≥40			1,689	1.9	
Paternal age at birth of child (years)	≤19			180	0.2	0.3
	20–24			3,497	4.1	
	25–29			19,167	22.3	
	30–34			33,725	39.3	
	35–39			20,734	24.1	
	≥40			8,563	10.1	
Maternal smoking during pregnancy	No			79,345	92.4	9.8
	Yes			6,521	7.6	
Paternal smoking during pregnancy	No			65,570	76.4	9.2
	Yes			20,296	23.6	
Maternal educational attainment	Incomplete upper 2° school			199	0.2	9.4
	Upper 2° school			1,527	1.8	
	High school/junior college			23,896	27.9	
	University/college, 4 years			36,309	42.3	
	University/college, >4 years			22,558	26.3	
	Other			1,377	1.6	
Parental language	Norwegian			76,521	89.1	11.3
	Other			9,345	10.9	
Offspring characteristics						
Gestational age (weeks)		39.8	2.0			0.4
Birth weight (g)		3,563	596			0.2
6-month BMI (kg/m^2^)		17.2	1.5			26.7
Age at 6-month BMI measurement (months)		5.8	0.5			26.4
1 year BMI (kg/m^2^)		17.0	1.4			37.7
Age at 1 year BMI measurement (years)		1.0	0.1			37.7
2-year BMI (kg/m^2^)		16.5	1.4			67.2
Age at 2-year BMI measurement (years)		2.0	0.2			66.8
3-year BMI (kg/m^2^)		16.1	1.5			58.5
Age at-3 year BMI measurement (years)		3.0	0.1			55.9
5-year BMI (kg/m^2^)		15.6	1.6			65.8
Age at 5-year BMI measurement (years)		5.2	0.3			63.7
8-year BMI (kg/m^2^)		16.1	2.0			51.3
Age at 8-year BMI measurement (years)		7.8	0.5			48.3

Statistics are for the sample used for linear regression analyses of birth weight (*n* = 85,866), aside from the other outcome variables, for which statistics are from the corresponding linear regression samples. Equivalent data for the 8-year BMI sample are presented in Information L in S1 Appendix. SD, standard deviations; WHO, World Health Organization.

^a^Percentage of the sample used for linear regression analyses of birth weight (i.e., denominator = 85,866).

^b^Percentage of 111,085 live born children with missing data for the relevant variable.

**Fig 2:**
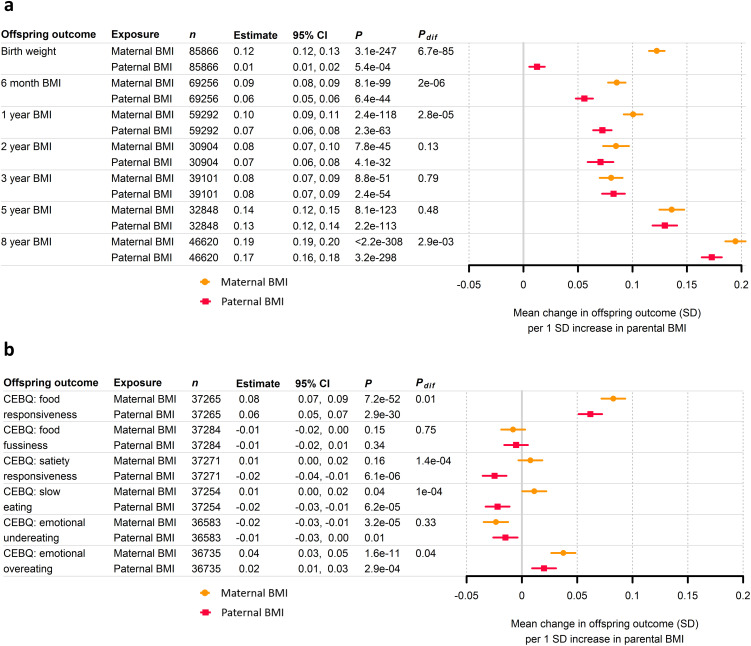
Linear associations of maternal and paternal BMI with offspring birth weight, child BMI and 8-year eating behaviours. Linear associations of maternal and paternal BMI with offspring outcomes. **a:** offspring birth weight and child BMI, **b**: offspring 8-year-old CEBQ eating behaviour traits, *P*: *P*-value testing the null hypothesis that regression coefficient is zero, *P*_dif_: *P*-value testing the null hypothesis that maternal and paternal regression coefficients are equal. In all models, we fitted covariates including the other parent’s BMI, and potential non-genetic confounders including maternal parity, parental and grandparental language group (as a proxy for ethnicity) and maternal and paternal characteristics (age, smoking during pregnancy, educational attainment and income). Error bars represent 95% confidence intervals.

Linear regression analyses provided strong statistical evidence that the association of maternal BMI with offspring birth weight is stronger than that of paternal BMI with offspring birth weight (Fig 2A). However, after birth the associations with offspring BMI converged, and the associations of maternal and paternal BMI with offspring 2 to 5-year BMI were similar. Although for 8-year BMI there was statistical evidence that the paternal association was slightly weaker, the difference was not large, and when we used unstandardised parental variables the paternal association was actually slightly stronger than the maternal association (S1 Table). These results were not markedly different when using offspring BMI predicted from a modelled growth curve (Information M in S1 Appendix), when substituting birth weight for ponderal index/BMI at birth and substituting child BMI for weight, when stratifying by offspring sex, or when additionally adjusting for gestational age (S1 Table). With respect to eating behaviour outcomes, both maternal and paternal BMI were positively associated with offspring food responsiveness and emotional overeating, and negatively associated with emotional undereating. Only paternal BMI was associated (negatively) with offspring satiety responsiveness and slow eating. Offspring 8-year BMI was associated with all eating behaviour outcomes except for emotional undereating, in the directions that would be expected from the behavioural susceptibility theory of obesity [34] (Information N in S1 Appendix). We did not observe large departures from log-linear relationships (Information G in S1 Appendix), and statistical interaction between maternal and paternal BMI was at most minor (the largest regression coefficient for the interaction term was −0.015 kg/m^2^) (Information O in S1 Appendix). The maternal BMI–paternal BMI correlation was 0.22 (95% CI [0.21, 0.23]; *p* < 0.001).

**Fig 3:**
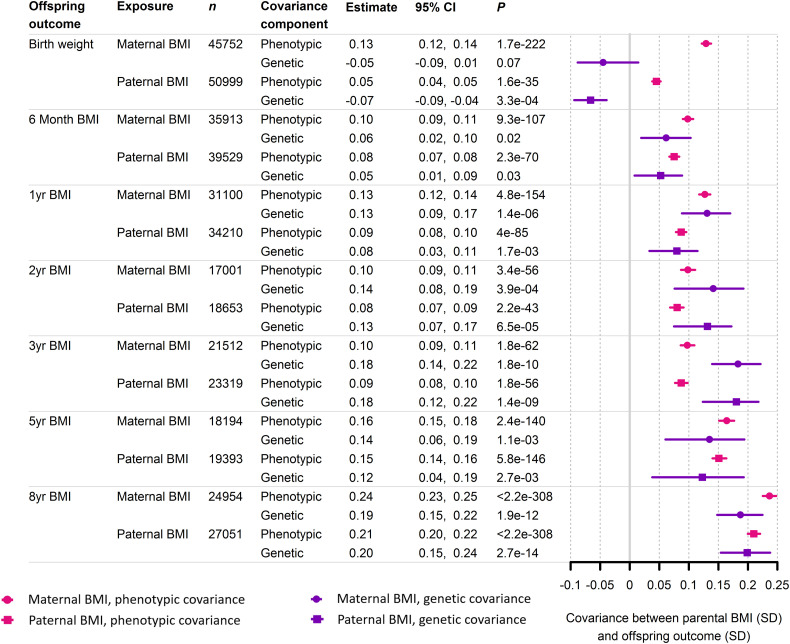
MCoTS SEM estimates of phenotypic and genetic covariance of parental BMI with offspring birth weight and BMI. **Phenotypic covariance** denotes the overall covariance between the exposure and outcome, **Genetic covariance** denotes the part of the phenotypic covariance that is due to genetic confounding, ***P***: *P*-value for phenotypic covariance calculated via a *z*-test using the standard error from bootstrapping the MCoTS model, *P*-value for genetic covariance calculated via a likelihood ratio chi-squared test for deterioration of model fit on constraint of the a1’ path coefficient to zero, confidence intervals for all covariances were calculated via a bias corrected bootstrap method, ***n***: number of offspring with outcome data available. Exposure variables were standardised to give unit variance and zero mean, and outcome variables were standardised within age and sex strata (or within only sex strata for birth weight), therefore covariances are approximately equal to Pearson’s correlation coefficients. Error bars represent 95% confidence intervals.

Table 2 shows the sample size available for MCoTS analyses, stratified by maternal and offspring relationship. The number of offspring with at least one non-missing observation included in the MCoTS analyses varied by outcome, from 50,999 to 17,001 for birth weight and 2-year BMI, respectively. The MCoTS results indicated that the positive phenotypic covariance between maternal BMI and offspring birth weight was not explained by genetic confounding, with genetic covariance estimates that were statistically indistinguishable from zero (Fig 3). The weak positive phenotypic covariance between paternal BMI and offspring birth weight was also not explained by genetic confounding. Surprisingly, there was statistical evidence for a small negative genetic covariance between paternal BMI and offspring birth weight, but this attenuated and became close to zero when, in exploratory analyses, we adjusted exposures and outcomes for potential confounders (including maternal BMI, paternal age and paternal income), suggesting that bias due to uncontrolled confounding may be the explanation (Information P in S1 Appendix).

**Table 2:** Number of families available for MCoTS analyses, stratified by parental and offspring relationship.

Relationship type	Relatedness coefficient	*n*			
		Total	Parental exposure data available^a^	Offspring BW data available^a^	Offspring 8-year BMI data available^a^
**Maternal BMI analyses**					
**Extended families (*n* = 16,292)**					
*n* stratified by mothers’ relatedness in each extended family					
MZ twin mother pair	1.00	61	60	61	48
DZ twin mother pair	0.50	34^b^	34	34	24
Full sibling mother pair	0.50	4,916	4,745	4,910	3,472
Maternal half-sibling mother pair	0.25	376	355	375	218
Unrelated sister-in-law mother pair	0.00	10,905	10,495	10,887	7,633
*n* offspring pairs linked to each mother^c^					
Full sibling offspring pair	0.50	4,538	4,477	4,535	3,154
Maternal half-sibling offspring pair	0.25	38	38	38	20
Unpaired (single) offspring	…	28,008	20,205	22,288	11,689
**Unpaired nuclear families (*n* = 7,339)**					
*n* offspring pairs linked to each mother					
MZ twin offspring pair	1.00	287	269	278	157
DZ twin offspring pair	0.50	738	698	735	379
Full sibling offspring pair	0.50	6,229	6,142	6,227	4,216
Maternal half-sibling offspring pair	0.25	85	81	85	37
**Paternal BMI analyses**					
**Extended families (*n* = 17,036)**					
*n* stratified by fathers’ relatedness in each extended family					
MZ twin father pair	1.00	27	27	27	24
DZ twin father pair	0.50	16^b^	16	16	13
Full sibling father pair	0.50	3,154	3,127	3,154	2,462
Paternal half-sibling father pair	0.25	171	168	171	128
Unrelated brother-in-law father pair	0.00	13,668	13,164	13,603	9,621
*n* offspring pairs linked to each father^c^					
Full sibling offspring pair	0.50	5,710	5,570	5,706	3,861
Paternal half-sibling offspring pair	0.25	28	28	28	22
Unpaired (single) offspring	…	28,334	22,207	25,046	12,605
**Unpaired nuclear families (*n* = 7,537)**					
*n* offspring pairs linked to each father					
MZ twin offspring pair	1.00	287	265	278	157
DZ twin offspring pair	0.50	951	854	924	453
Full sibling offspring pair	0.50	6,229	6,152	6,227	4,216
Paternal half-sibling offspring pair	0.25	70	67	69	39

^a^Number of relative pairs for which at least one member had exposure/outcome data available (the numbers given in Figs 3 and 4 for individual offspring with available data for each outcome are therefore greater).

^b^Parent-generation relatedness was modelled between same-sex DZ twins/siblings only, therefore opposite-sex DZ twins/siblings were treated as unrelated siblings-in-law.

^c^MZ and DZ twins in the offspring generation were only retained for singleton parents. MZ, monozygotic (identical) twins; DZ, dizygotic (non-identical) twins.

**Fig 4:**
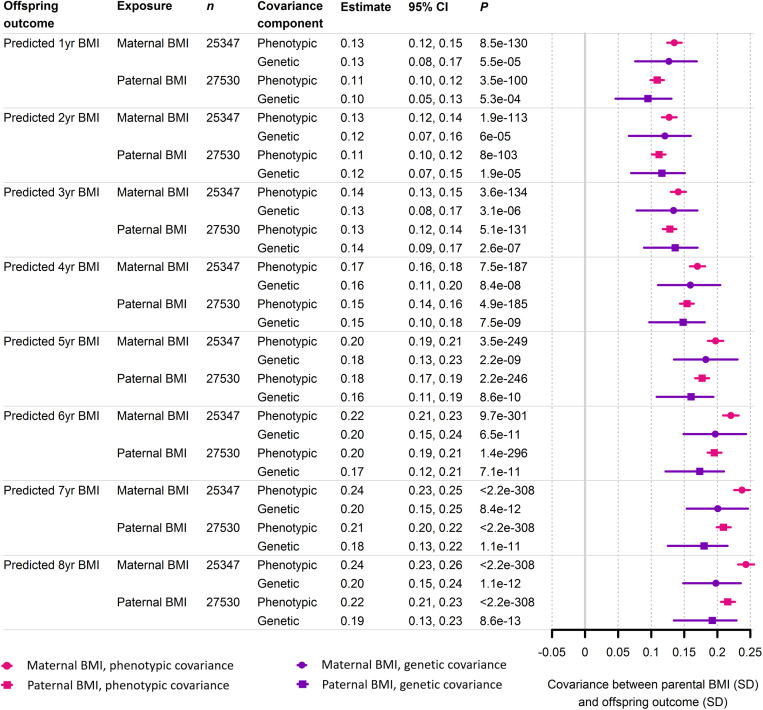
MCoTS SEM estimates of phenotypic and genetic covariance of parental BMI with offspring predicted BMI. **Phenotypic covariance** denotes the overall covariance between the exposure and outcome, **Genetic covariance** denotes the part of the phenotypic covariance that is due to genetic confounding, ***P***: *P*-value for phenotypic covariance calculated via a *z*-test using the standard error from bootstrapping the MCoTS model, *P*-value for genetic covariance calculated via a likelihood ratio chi-squared test for deterioration of model fit on constraint of the a1’ path coefficient to zero, confidence intervals for all covariances were calculated via a bias corrected bootstrap method, ***n***: number of offspring with outcome data available. Exposure variables were standardised to give unit variance and zero mean, and outcome variables were standardised within age and sex strata (or within only sex strata for birth weight), therefore covariances are approximately equal to Pearson’s correlation coefficients. Error bars represent 95% confidence intervals.

From age 6 months onwards, genetic covariance estimates became positive and increased in magnitude, such that for offspring 8-year BMI, genetic confounding explained 79% (0.19/0.24 * 100; 95% CI [62, 95]; *p* = 1.9 × 10^−12^) of the covariance with maternal BMI and 94% (0.20/0.21 * 100; 95% CI [72, 113]; *p* = 2.7 × 10^−14^) of the covariance with paternal BMI (Fig 3). For some outcomes, genetic covariance estimates were greater than the phenotypic covariance estimates. This was potentially due to sampling error, whereby the genetic covariance estimated in the sample was greater than the true genetic covariance in the population; in such cases the 95% CIs for genetic covariance estimates usually included the phenotypic covariance point estimate. Genetic confounding explained a high and stable proportion of the phenotypic covariance with predicted BMI from age 1 to 8 years (Fig 4). Results did not appreciably differ when we fitted ACE or AE models instead of the primary ADE model in the parent generation (S2 Table), when birth weight was substituted for ponderal index/BMI at birth and child BMI was substituted for weight (S2 Table and Information Q in S1 Appendix), or when birth weight models were refit without offspring-generation twins (results available from the authors on request). Although sex stratified models were underpowered, they provided no evidence for large sex differences in estimates (S2 Table). MCoTS models for eating behaviour outcomes were underpowered and uninformative (S2 Table and Information R in S1 Appendix). Full MCoTS results including model fit statistics and estimated variance components for parental and offspring phenotypes are presented in S2 Table.

## Discussion

We triangulated evidence from two analytic approaches applied to a large European birth cohort, to infer the mechanisms underlying associations between parental BMI and offspring birth weight, BMI until 8 years and 8-year eating behaviour. There were not large differences in the magnitude of the associations of maternal and paternal BMI with offspring BMI beyond early childhood, suggesting confounding within families as the most parsimonious explanation for such associations. Such confounding could involve environmental (non-genetic) factors shared within families, or could instead be due to the inheritance of parental genetic alleles by the offspring (genetic confounding). The latter mechanism was supported by our MCoTS analyses, which indicated that the covariance between parental BMI and offspring BMI from age 6 months to 8 years may primarily be due to genetic confounding. Importantly, the present study in isolation cannot provide definitive conclusions about the causes of parent–child adiposity associations, and must be interpreted in light of previous studies that have deployed orthogonal approaches.

We have previously used genomic restricted maximum likelihood (GCTA-GREML) to explore whether intergenerational BMI associations could be due to genetic confounding [26]. Those analyses investigated whether mother-offspring BMI covariance was explained by a set of ~8 million imputed common genetic variants, in nominally unrelated individuals from 3 European birth cohorts. In contrast, the present MCoTS analyses investigated confounding involving all genetic variants, using expected relatedness inferred via quantitative genetic theory in a pedigree of close relatives. GCTA-GREML indicated that for maternal BMI, imputed variants explained 43% (95% CI [15, 72]) of the covariance with offspring 15-year BMI, with a similar estimate for 10-year BMI. This is highly consistent with the present MCoTS estimates: 79% (95% CI [62, 95]) of the covariance between maternal BMI and offspring 8-year BMI was explained by genetic confounding. MCoTS estimates were somewhat higher than those from GCTA-GREML, which is possible given that GCTA-GREML uses a set of measured genetic variants whereas MCoTS infers the effects of all variants. Furthermore, both GCTA-GREML and MCoTS indicated that the covariance between maternal BMI and offspring birth weight was unlikely to be due to genetic confounding. The high concordance of results between the two methods, which make different assumptions and were applied to different cohorts, provides strong evidence that genetic confounding may be a major driver of associations between parental BMI and offspring adiposity in late childhood.

Our results are consistent with previous maternal–paternal comparison, MR and sibling studies (several of which share authors with the present study), which have not supported a large causal effect of maternal BMI on offspring childhood adiposity [21,22,24,25]. However, sibling studies do provide a degree of support for potential causal effects of more extreme maternal metabolic dysregulation (for example, maternal diabetes and severe obesity) on offspring adiposity beyond birth [7,22], putatively mediated via epigenetic mechanisms [39]. Taken together, this suggests that putative causal effects observed in animal models of developmental overnutrition [10,17] should be interpreted cautiously and do not necessarily occur in humans. The association between maternal BMI and offspring size/adiposity at birth has been invoked in the literature to argue that maternal adiposity has a causal effect on offspring adiposity beyond birth [40]. Indeed, MR and sibling studies [21,27,41,42] support a causal effect of maternal BMI on offspring birth size. However, the present results, alongside previous studies [21,25–27,41], suggest that the causes of weight at birth are somewhat different from the causes of BMI in later childhood.

We observed some associations between parental BMI and offspring obesity-related eating behaviours assessed via the CEBQ questionnaire. Previous studies in smaller samples have found similar associations, albeit somewhat inconsistently [20,43]. In particular, we found that greater maternal and paternal BMI were associated with increased scores on the CEBQ food responsiveness and emotional overeating scales, and a reduced score on the emotional undereating scale. Offspring satiety responsiveness and slow eating were only associated with paternal BMI; however, we cannot exclude measurement error as an explanation for the absent maternal associations because the CEBQ questionnaire was completed by mothers and is inherently subjective. Given our maternal–paternal comparison results it is plausible that child appetite-related eating behaviours mediate the genetically driven association between parent and child BMI. However, it was not possible to confirm this because our MCoTS analyses were underpowered for eating behaviours.

Our results may have important public health implications, when considered alongside prior evidence. Maternal BMI may be unlikely to have a large causal effect on child BMI beyond birth, although a small causal effect remains plausible, potentially mediated via maternal glycaemia during pregnancy. Any causal effect of paternal BMI on offspring childhood BMI is likely to be similar to or smaller than that of maternal BMI. Consequently, reductions in the BMI of either parent before pregnancy may be unlikely to cause large reductions in childhood adiposity. It is possible that interventions targeting parental BMI reduction could influence childhood adiposity via parental lifestyle changes that persist after birth and affect the offspring’s environment. There may also be complex relationships between elements of the social environment including socioeconomic position, and aspects of genetic and environmental obesity risk. However, whether preconceptional interventions are the optimal approach for preventing childhood obesity requires further evaluation in light of evidence from the present study. Importantly, women considering pregnancy should still be advised and supported to maintain a healthy weight, because there is good evidence that maternal obesity in pregnancy causes adverse perinatal outcomes in the mother and offspring [44]. Furthermore, our results do not support genetic determinism, because even children who inherit increased genetic obesity predisposition from their parents could express those genes differentially in different environments.

Our study has important strengths. We have analysed data from a large prospective birth cohort, enabling precise estimation of the associations between parental BMI assessed during pregnancy and offspring outcomes in mid-childhood. We leveraged a pedigree involving twin and sibling relationships in both the parental and offspring generations, to partition parent-offspring phenotypic covariance via an MCoTS SEM that is rooted in quantitative genetics theory. We gained increased power over a conventional children of twins model by including non-twin siblings in the parent and offspring generations, and up to two children for each parent. The MCoTS model includes genetic covariances between siblings in the parent and offspring generations and between cousins in the offspring generation, as well as parent–child and avuncular genetic covariances. Because all these covariances are genetically informative, statistical power is dramatically increased over standard children of twins models [36]. Finally, we used methods which make different assumptions to previous MR, GCTA-GREML and within-sibling analyses [21,22,24–26], facilitating opportunities for triangulation of evidence. We also acknowledge potential limitations of this study. First, because the MCoTS model cannot simultaneously estimate dominance genetic effects and common environmental effects, we fitted an ADE model in the parent generation, which assumes that common environmental effects are absent. This assumption is supported by classic and extended twin studies of adult BMI [15] and the similarity of our findings when we used ACE or AE models in the parent generation (S2 Table). Second, our MCoTS analyses assume that phenotypic associations between log parent BMI and offspring outcomes are linear. In our data there were mild deviations from log-linearity for the associations with some offspring outcomes at younger ages (birth to three years) (Information G in S1 Appendix). However, the associations with offspring BMI at older ages were approximately linear. Third, although our maternal–paternal comparison analyses were mutually adjusted for the other parent’s BMI (with the aim of maximising control of confounding), our MCoTS analyses did not account for assortative mating. However, we do not expect this to have had a large impact on our results because spousal phenotypic correlations for BMI, including in the current study, are relatively weak [21,45]. Fourth, the MCoTS model does not account for gene by environment interaction, and if interactions exist between the additive genetic and common environmental variance components our results could overestimate genetic confounding. We believe any such bias is likely to be small; however, because although the behavioural susceptibility theory of obesity predicts widespread gene by environment interaction for obesity genetic variants, extended twin family design data suggest common environmental effects are negligible for adult BMI [15], and such common environmental effects are the likely source of potential bias for MCoTS models. Fifth, the residual covariance estimated by the MCoTS model will not be indicative of the true causal effect of parental BMI, to the extent that residual confounding affects associations between parental BMI and offspring outcomes. It is likely that the negative residual covariance estimate for paternal BMI and offspring birth weight reflects residual confounding, particularly as this estimate attenuated on adjustment for potential confounders (Information O in S1 Appendix). Sixth, our maternal–paternal comparisons did not account for non-paternity, which could weaken paternal associations. However, a previous simulation study showed that for a maternal–paternal comparison analysis using MoBa data with follow-up to age three years, results would have changed little with non-paternity rates of up to 10% [25]. Seventh, we used BMI calculated predominantly with measurements from the child’s health card (ages up 3 years), or measurements carried out by the parents (from 5 years onwards), which could have introduced bias due to measurement error since BMI is an imperfect proxy measure for adiposity. Despite this, BMI is highly correlated with more direct adiposity measures in childhood [46]. Eighth, MoBa has a participation rate of 41% and there has been attrition over follow-up [32]. Although we cannot definitively rule out selection bias, there were no large differences in the distributions of key variables between our baseline and 8 year follow up samples (Information L in S1 Appendix). It therefore seems unlikely that our results are subject to selection bias of sufficient magnitude to alter our conclusions. Ninth, we analysed offspring BMI at ages up to eight years, and since the maternal BMI-offspring overweight/obesity association strengthens in adolescence [47], we cannot draw conclusions about the importance of genetic confounding for child BMI beyond eight years. Despite this, our previous GCTA-GREML analyses suggest that the degree of genetic confounding might actually increase into adulthood [26]. Lastly, we have studied a Norwegian population born between 1999 and 2009, which has relatively high obesity prevalence and income per capita in international terms, and it would be beneficial to replicate our analyses in other settings.

In summary, we have shown that in a Norwegian population the linear association between parental BMI around the time of pregnancy and offspring BMI from age 6 months to 8 years may primarily be due to genetic confounding. If our results are replicated in other populations, particularly in cohorts exposed to higher obesity prevalence in early life, this finding, taken with those from previous studies using alternative methods, may suggest that neither the mothers’ nor fathers’ pre-pregnancy BMI has a large causal effect on childhood BMI. This could imply that any hypothetical intervention that successfully reduced parental BMI before pregnancy, without altering the offspring’s postnatal environment, might be insufficient to achieve large reductions in the offspring’s childhood obesity risk.

## Supporting information

S1 STROBE Checklist STrengthening the Reporting of OBservational studies in Epidemiology Checklist. Downloaded from https://www.strobe-statement.org/. *von Elm E, Altman DG, Egger M, Pocock SJ, Gøtzsche PC, Vandenbroucke JP; STROBE Initiative. The Strengthening the Reporting of Observational Studies in Epidemiology (STROBE) statement: guidelines for reporting observational studies. PLoS Med. 2007 Oct 16;4(10):e296. PMID: 17941714.* This checklist is licensed under the Creative Commons Attribution 4.0 International License (CC BY 4.0; https://creativecommons.org/licenses/by/4.0/).
(DOCX)

S1 Appendix Supplementary information. Supplementary information A: Sample selection flowchart. Supplementary information B: Anthropometric data cleaning and growth curve fitting. Supplementary information C: Covariate data. Supplementary information D: Linear mixed model to account for non-independence between siblings. Supplementary information E: *z*-test for the difference in maternal and paternal associations. Supplementary information F: Summary statistics for transformed and untransformed exposure and outcome variables. Supplementary information G: Tests for non-linear associations between exposures and outcomes. Supplementary information H: Multiple Children of Twins and Siblings (MCoTS) model. Supplementary information I: MCoTS model with ACE partition of parental exposure. Supplementary information J: MCoTS model with AE partition of parental exposure. Supplementary information K: Liability threshold model for untransformed CEBQ outcomes. Supplementary information L: Differences in participant characteristics between baseline and 8-year-old sample. Supplementary information M: Linear associations between parental BMI and offspring predicted BMI. Supplementary information N: Linear associations between offspring 8-year BMI and CEBQ outcomes. Supplementary information O: Statistical interaction between maternal and paternal BMI. Supplementary information P: MCoTS results for the association of parental BMI with offspring birth weight, adjusted for potential confounders. Supplementary information Q: MCoTS results for the association of parental BMI with offspring weight, BMI and ponderal index at birth. Supplementary information R: MCoTS results for the association of parental BMI with offspring eating behaviour (CEBQ) traits.
(PDF)

S1 Table Full linear regression results for associations between exposures and outcomes. (XLSX)

S2 Table Full MCoTS results including model fit statistics and estimated variance components for parental and offspring phenotypes. (XLSX)

## Abbreviations:

BMI: body mass index
BW: birth weight
CEBQ: Child Eating Behaviour Questionnaire
MBRN: Medical Birth Registry of Norway
MCoTS: Multiple Children of Twins and Siblings
MoBa: Norwegian Mother, Father and Child Cohort Study
MR: Mendelian randomisation
MZ: monozygotic
SD: standard deviation
SEM: structural equation model

## References

[1] Ling J, Chen S, Zahry NR, Kao T-SA. Economic burden of childhood overweight and obesity: a systematic review and meta-analysis. Obes Rev. 2023;24(2):e13535. doi: 10.1111/obr.13535 36437105 PMC10078467

[2] Kerr JA, Patton GC, Cini KI, Abate YH, Abbas N, Abd Al Magied AHA. Global, regional, and national prevalence of child and adolescent overweight and obesity, 1990–2021, with forecasts to 2050: a forecasting study for the Global Burden of Disease Study 2021. Lancet. 2025;405(10481):785–812. 40049185 10.1016/S0140-6736(25)00397-6 PMC11920006

[3] Halilagic A, Moschonis G. The effect of growth rate during infancy on the risk of developing obesity in childhood: a systematic literature review. Nutrients. 2021;13(10):3449. doi: 10.3390/nu13103449 34684450 PMC8537274

[4] Zhang J, Clayton GL, Overvad K, Olsen A, Lawlor DA, Dahm CC. Body mass index in parents and their adult offspring: a systematic review and meta-analysis. Obes Rev. 2024;25(1):e13644. doi: 10.1111/obr.13644 37783229 PMC10909538

[5] Hanson M, Barker M, Dodd JM, Kumanyika S, Norris S, Steegers E, et al. Interventions to prevent maternal obesity before conception, during pregnancy, and post partum. Lancet Diabetes Endocrinol. 2017;5(1):65–76. doi: 10.1016/S2213-8587(16)30108-5 27743974

[6] Heslehurst N, Vieira R, Akhter Z, Bailey H, Slack E, Ngongalah L, et al. The association between maternal body mass index and child obesity: a systematic review and meta-analysis. PLoS Med. 2019;16(6):e1002817. doi: 10.1371/journal.pmed.1002817 31185012 PMC6559702

[7] Lawlor DA. The Society for Social Medicine John Pemberton Lecture 2011. Developmental overnutrition—an old hypothesis with new importance?. Int J Epidemiol. 2013;42(1):7–29. 23508404 10.1093/ije/dys209

[8] Godfrey KM, Reynolds RM, Prescott SL, Nyirenda M, Jaddoe VWV, Eriksson JG, et al. Influence of maternal obesity on the long-term health of offspring. Lancet Diabetes Endocrinol. 2017;5(1):53–64. doi: 10.1016/S2213-8587(16)30107-3 27743978 PMC5245733

[9] Sharp GC, Lawlor DA. Paternal impact on the life course development of obesity and type 2 diabetes in the offspring. Diabetologia. 2019;62(10):1802–10. doi: 10.1007/s00125-019-4919-9 31451867 PMC6731203

[10] Lane M, Zander-Fox DL, Robker RL, McPherson NO. Peri-conception parental obesity, reproductive health, and transgenerational impacts. Trends Endocrinol Metab. 2015;26(2):84–90. doi: 10.1016/j.tem.2014.11.005 25523615

[11] McPherson NO, Fullston T, Aitken RJ, Lane M. Paternal obesity, interventions, and mechanistic pathways to impaired health in offspring. Ann Nutr Metab. 2014;64(3–4):231–8. doi: 10.1159/000365026 25300265

[12] Larqué E, Labayen I, Flodmark C-E, Lissau I, Czernin S, Moreno LA, et al. From conception to infancy – early risk factors for childhood obesity. Nat Rev Endocrinol. 2019;15(8):456–78. doi: 10.1038/s41574-019-0219-1 31270440

[13] Fleming TP, Watkins AJ, Velazquez MA, Mathers JC, Prentice AM, Stephenson J, et al. Origins of lifetime health around the time of conception: causes and consequences. Lancet. 2018;391(10132):1842–52. doi: 10.1016/S0140-6736(18)30312-X 29673874 PMC5975952

[14] Billah MM, Khatiwada S, Morris MJ, Maloney CA. Effects of paternal overnutrition and interventions on future generations. Int J Obes (Lond). 2022;46(5):901–17. doi: 10.1038/s41366-021-01042-7 35022547 PMC9050512

[15] Maes HH, Neale MC, Eaves LJ. Genetic and environmental factors in relative body weight and human adiposity. Behav Genet. 1997;27(4):325–51. doi: 10.1023/a:1025635913927 9519560

[16] Mahmood L, Flores-Barrantes P, Moreno LA, Manios Y, Gonzalez-Gil EM. The influence of parental dietary behaviors and practices on children’s eating habits. Nutrients. 2021;13(4):1138. doi: 10.3390/nu13041138 33808337 PMC8067332

[17] Friedman JE. Developmental programming of obesity and diabetes in mouse, monkey, and man in 2018: where are we headed?. Diabetes. 2018;67(11):2137–51. doi: 10.2337/dbi17-0011 30348820 PMC6198344

[18] Rasmussen JM, Thompson PM, Entringer S, Buss C, Wadhwa PD. Fetal programming of human energy homeostasis brain networks: issues and considerations. Obes Rev. 2022;23(3):e13392. doi: 10.1111/obr.13392 34845821 PMC10308600

[19] Webber L, Hill C, Saxton J, Van Jaarsveld CHM, Wardle J. Eating behaviour and weight in children. Int J Obes (Lond). 2009;33(1):21–8. doi: 10.1038/ijo.2008.219 19002146 PMC2817450

[20] Albuquerque G, Severo M, Oliveira A. Early life characteristics associated with appetite-related eating behaviors in 7-year-old children. J Pediatr. 2017;180:38-46.e2. doi: 10.1016/j.jpeds.2016.09.011 27769552

[21] Bond TA, Richmond RC, Karhunen V, Cuellar-Partida G, Borges MC, Zuber V, et al. Exploring the causal effect of maternal pregnancy adiposity on offspring adiposity: mendelian randomisation using polygenic risk scores. BMC Med. 2022;20(1):34. doi: 10.1186/s12916-021-02216-w 35101027 PMC8805234

[22] Lawlor DA, Lichtenstein P, Långström N. Association of maternal diabetes mellitus in pregnancy with offspring adiposity into early adulthood: sibling study in a prospective cohort of 280,866 men from 248,293 families. Circulation. 2011;123(3):258–65. doi: 10.1161/CIRCULATIONAHA.110.980169 21220735 PMC4440894

[23] Branum AM, Parker JD, Keim SA, Schempf AH. Prepregnancy body mass index and gestational weight gain in relation to child body mass index among siblings. Am J Epidemiol. 2011;174(10):1159–65. doi: 10.1093/aje/kwr250 21984656

[24] Patro B, Liber A, Zalewski B, Poston L, Szajewska H, Koletzko B. Maternal and paternal body mass index and offspring obesity: a systematic review. Ann Nutr Metab. 2013;63(1–2):32–41. doi: 10.1159/000350313 23887153

[25] Fleten C, Nystad W, Stigum H, Skjaerven R, Lawlor DA, Davey Smith G, et al. Parent-offspring body mass index associations in the Norwegian Mother and Child Cohort Study: a family-based approach to studying the role of the intrauterine environment in childhood adiposity. Am J Epidemiol. 2012;176(2):83–92. doi: 10.1093/aje/kws134 22771730 PMC3493198

[26] Bond TA, Karhunen V, Wielscher M, Auvinen J, Männikkö M, Keinänen-Kiukaanniemi S, et al. Exploring the role of genetic confounding in the association between maternal and offspring body mass index: evidence from three birth cohorts. Int J Epidemiol. 2020;49(1):233–43. doi: 10.1093/ije/dyz095 31074781 PMC7245052

[27] Tyrrell J, Richmond RC, Palmer TM, Feenstra B, Rangarajan J, Metrustry S, et al. Genetic evidence for causal relationships between maternal obesity-related traits and birth weight. JAMA. 2016;315(11):1129–40. doi: 10.1001/jama.2016.1975 26978208 PMC4811305

[28] Elks CE, den Hoed M, Zhao JH, Sharp SJ, Wareham NJ, Loos RJF, et al. Variability in the heritability of body mass index: a systematic review and meta-regression. Front Endocrinol (Lausanne). 2012;3:29. doi: 10.3389/fendo.2012.00029 22645519 PMC3355836

[29] Silventoinen K, Jelenkovic A, Sund R, Hur Y-M, Yokoyama Y, Honda C, et al. Genetic and environmental effects on body mass index from infancy to the onset of adulthood: an individual-based pooled analysis of 45 twin cohorts participating in the COllaborative project of Development of Anthropometrical measures in Twins (CODATwins) study. Am J Clin Nutr. 2016;104(2):371–9. doi: 10.3945/ajcn.116.130252 27413137 PMC4962159

[30] Yang J, Bakshi A, Zhu Z, Hemani G, Vinkhuyzen AAE, Lee SH, et al. Genetic variance estimation with imputed variants finds negligible missing heritability for human height and body mass index. Nat Genet. 2015;47(10):1114–20. doi: 10.1038/ng.3390 26323059 PMC4589513

[31] Vogelezang S, Bradfield JP, Ahluwalia TS, Curtin JA, Lakka TA, Grarup N, et al. Novel loci for childhood body mass index and shared heritability with adult cardiometabolic traits. PLoS Genet. 2020;16(10):e1008718. doi: 10.1371/journal.pgen.1008718 33045005 PMC7581004

[32] Magnus P, Birke C, Vejrup K, Haugan A, Alsaker E, Daltveit AK. Cohort profile update: the Norwegian Mother and Child Cohort Study (MoBa). Int J Epidemiol. 2016. doi: dyw029 10.1093/ije/dyw029 27063603

[33] Irgens LM. The Medical Birth Registry of Norway. Epidemiological research and surveillance throughout 30 years. Acta Obstet Gynecol Scand. 2000;79(6):435–9. doi: 10.1080/j.1600-0412.2000.079006435.x 10857866

[34] Wardle J, Guthrie CA, Sanderson S, Rapoport L. Development of the children’s eating behaviour questionnaire. J Child Psychol Psychiatry. 2001;42(7):963–70. doi: 10.1111/1469-7610.00792 11693591

[35] R Core Team. R: A language and environment for statistical computing. Vienna, Austria: R Foundation for Statistical Computing; 2020.

[36] McAdams TA, Hannigan LJ, Eilertsen EM, Gjerde LC, Ystrom E, Rijsdijk FV. Revisiting the children-of-twins design: improving existing models for the exploration of intergenerational associations. Behav Genet. 2018;48(5):397–412. doi: 10.1007/s10519-018-9912-4 29961153 PMC6097723

[37] Lunde A, Melve KK, Gjessing HK, Skjaerven R, Irgens LM. Genetic and environmental influences on birth weight, birth length, head circumference, and gestational age by use of population-based parent-offspring data. Am J Epidemiol. 2007;165(7):734–41. doi: 10.1093/aje/kwk107 17311798

[38] Neale MC, Hunter MD, Pritikin JN, Zahery M, Brick TR, Kirkpatrick RM, et al. OpenMx 2.0: extended structural equation and statistical modeling. Psychometrika. 2016;81(2):535–49. 25622929 10.1007/s11336-014-9435-8 PMC4516707

[39] Guénard F, Deshaies Y, Cianflone K, Kral JG, Marceau P, Vohl M-C. Differential methylation in glucoregulatory genes of offspring born before vs. after maternal gastrointestinal bypass surgery. Proc Natl Acad Sci U S A. 2013;110(28):11439–44. doi: 10.1073/pnas.1216959110 23716672 PMC3710842

[40] Catalano PM, Shankar K. Obesity and pregnancy: mechanisms of short term and long term adverse consequences for mother and child. BMJ. 2017;356:j1. doi: 10.1136/bmj.j1 28179267 PMC6888512

[41] Chen J, Bacelis J, Sole-Navais P, Srivastava A, Juodakis J, Rouse A, et al. Dissecting maternal and fetal genetic effects underlying the associations between maternal phenotypes, birth outcomes, and adult phenotypes: a mendelian-randomization and haplotype-based genetic score analysis in 10,734 mother-infant pairs. PLoS Med. 2020;17(8):e1003305. doi: 10.1371/journal.pmed.1003305 32841251 PMC7447062

[42] Villamor E, Cnattingius S. Interpregnancy weight change and risk of adverse pregnancy outcomes: a population-based study. Lancet. 2006;368(9542):1164–70. doi: 10.1016/S0140-6736(06)69473-7 17011943

[43] Boswell N, Byrne R, Davies PSW. Eating behavior traits associated with demographic variables and implications for obesity outcomes in early childhood. Appetite. 2018;120:482–90. doi: 10.1016/j.appet.2017.10.012 29024677

[44] Borges MC, Clayton G, Freathy RM, Felix JF, Fernández-Sanlés A, Soares AG, et al. Integrating multiple lines of evidence to assess the effects of maternal BMI on pregnancy and perinatal outcomes in up to 497,932 women. medRxiv. 2022:2022.07.22.22277930. 10.1186/s12916-023-03167-0 PMC10823651 38281920

[45] Horwitz TB, Keller MC. A comprehensive meta-analysis of human assortative mating in 22 complex traits. bioRxiv. 2022. doi: 2022.03.19.484997

[46] Bell JA, Carslake D, O’Keeffe LM, Frysz M, Howe LD, Hamer M, et al. Associations of body mass and fat indexes with cardiometabolic traits. J Am Coll Cardiol. 2018;72(24):3142–54. 30545453 10.1016/j.jacc.2018.09.066 PMC6290112

[47] Voerman E, Santos S, Patro Golab B, Amiano P, Ballester F, Barros H, et al. Maternal body mass index, gestational weight gain, and the risk of overweight and obesity across childhood: an individual participant data meta-analysis. PLoS Med. 2019;16(2):e1002744. doi: 10.1371/journal.pmed.1002744 30742624 PMC6370184

